# Kidney Metastasis From Adenoid Cystic Carcinoma of the Submandibular Gland: A Case Report and Literature Review

**DOI:** 10.7759/cureus.107848

**Published:** 2026-04-27

**Authors:** Renda Alhabib, Chaker Zaidi, Nuran Baabbad, Ibrahim Alotain

**Affiliations:** 1 Radiation Oncology, King Fahad Specialist Hospital, Dammam, SAU

**Keywords:** adenoid cystic carcinoma, case report, rare metastasis, renal metastases, submandibular gland

## Abstract

Adenoid cystic carcinoma (ACC) of the salivary glands is characterized by perineural invasion, local recurrence, and distant metastasis, predominantly to the lungs; however, unusual metastatic sites have also been reported.

Renal metastasis from primary ACC is clinically rare. To the best of our knowledge, there have been only 13 cases of ACC metastasizing to the kidney. Here, we present an additional case of left renal metastases originating from a left submandibular gland ACC.

In this report, we describe a 38-year-old male who developed left renal metastases 30 months after completing postoperative radiotherapy for ACC of the left submandibular gland. We also provide a review of the literature on renal metastases from ACC, highlighting its rarity, clinical presentation, radiological findings, and management strategies.

## Introduction

Adenoid cystic carcinoma (ACC) is an uncommon malignancy, representing approximately 1% of all head and neck cancers [1]. Despite its rarity, ACC is clinically significant due to its unique biology marked by slow growth, frequent perineural invasion, and a tendency for late recurrence and distant metastasis.

ACC predominantly originates in the major and minor salivary glands, with the parotid and submandibular glands being the most frequent sites. However, it may also originate, though less commonly, in other anatomical locations, including the lacrimal glands, trachea, breasts, and lungs [2].

Despite advances in treatment modalities, the 10-year survival rate for ACC remains poor, at less than 50%, regardless of the histologic grade or initial stage of the disease [3].

The tumor is particularly challenging due to its high rates of recurrence and distant metastasis [4]. While renal metastasis is frequently observed in cancers of the lung, breast, and other organs [5], renal metastasis originating from ACC is exceptionally rare, with only 13 documented cases in the medical literature to date [1,6-17].

Bilateral renal metastases are even more uncommon, and, to the best of our knowledge, only one prior case has been reported, which originated from the lung [6].

In this case report, we present the detailed clinical course of a 38-year-old male patient who developed left renal metastases from ACC of the left submandibular gland. This case is particularly remarkable not only because of the rarity of renal involvement but also due to the patient’s relatively young age and the aggressive nature of the disease.

The aim of this report is to contribute to the limited body of knowledge on renal metastases in ACC, highlight the diagnostic and therapeutic challenges associated with this condition, and emphasize the importance of follow-up in patients with ACC, given its propensity for recurrence and metastasis.

## Case presentation

A 38-year-old male presented with a one-year history of a painless, progressively enlarging 2 × 2 cm swelling in the left submandibular gland. The patient reported no associated symptoms such as difficulty swallowing, breathing, or weight loss. He was a heavy smoker (50 pack-years) and had no family history of malignancy.

Physical examination revealed a hard, fixed, non-tender 2 × 2 cm mass in the left submandibular gland. There was no tongue deviation or cervical lymphadenopathy. Given the clinical presentation, a biopsy was performed, and the histopathology findings confirmed the diagnosis of solid-pattern ACC.

A CT scan of the chest, abdomen, and pelvis was performed to rule out distant metastases, and the results were negative for any metastasis.

The patient underwent surgical excision of the left submandibular mass. Pathology findings revealed a high-grade ACC of 2.5 cm with extra-parenchymal extension, positive surgical margins, positive perineural invasion (PNI), and negative lymphovascular invasion (LVI). The tumor was staged as pT3, pNx, as only lymph node sampling was performed, collecting three lymph nodes that were negative (0/3).

Following the surgery, a CT scan showed a mild soft tissue density (1.5 cm) at the surgical bed with a tiny hyperdense/enhancing focus, potentially indicative of residual disease. The right submandibular and parotid glands appeared unremarkable, and multiple bilateral small cervical lymph nodes were noted, though none met the CT size criteria for metastasis.

**Figure 1:**
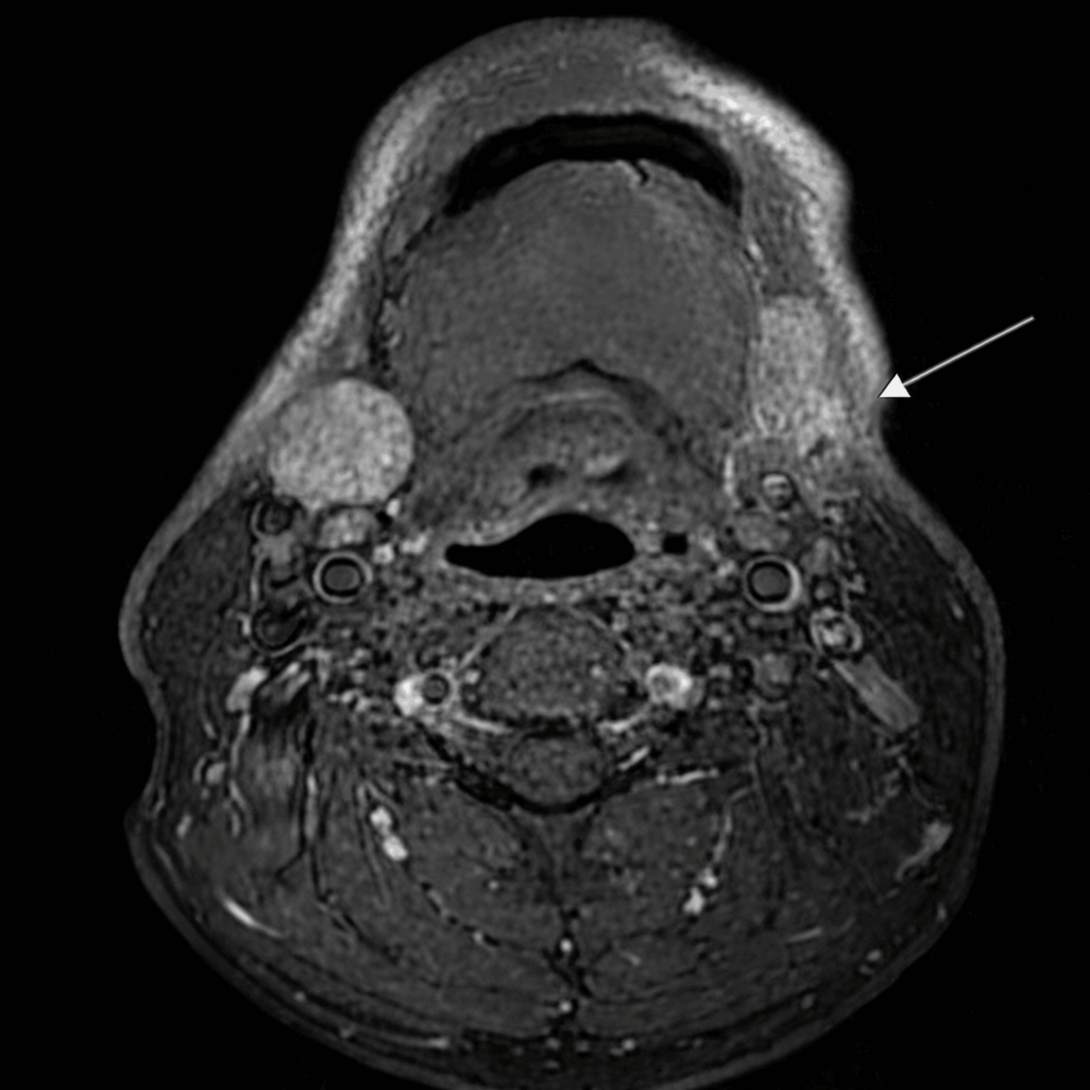
Axial T1 MRI of the neck with contrast showing resection of the left submandibular gland tumor with ill-defined soft tissue infiltration and heterogeneous enhancement in the surgical bed measuring approximately 2.4 x 2.5 x 2.1 cm, involving the cutaneous and subcutaneous region.

MRI showed postoperative changes obscuring the surgical bed, making accurate assessment of residual or recurrent tumor impossible (Figure 1). A suspicious left level Ib lymph node was noted, which prompted further evaluation.

The case was discussed at the multidisciplinary head and neck tumor board, and it was decided to proceed with a second-look surgery.

The patient underwent a second-look surgery of the left submandibular bed mass and left neck dissection. Pathology findings confirmed the presence of multifocal solid-pattern ACC, with LVI. Additionally, the tumor showed involvement of the deep, inferior, and superior margins. Lateral and medial margins were 1 mm from the tumor. Of the 34 lymph nodes sampled, two were positive for metastasis. The tumor was also noted to be HER-2 negative.

Subsequently, the patient received adjuvant radiotherapy (66 Gy in 33 fractions) to the primary tumor bed and ipsilateral neck nodal levels at risk (levels I-III). The clinical target volume was extended to include potential perineural pathways along the involved nerve up to the skull base, given the known propensity of ACC for perineural spread. No concurrent chemotherapy was administered. At three-month follow-up after completion of radiotherapy, CT imaging of the neck showed no evidence of residual or recurrent disease.

Ten months later, the patient developed a new mass in the pre-auricular/masseteric region. Fine-needle aspiration (FNA) confirmed recurrent ACC. A staging workup, including CT of the chest and PET-CT, was performed and showed no evidence of distant metastasis.

He underwent revision surgery consisting of left submandibular bed mass excision and level 5 lymph node dissection. The pathology findings revealed residual solid-type ACC (1 cm in size), with the tumor located 1 mm from the nearest resection margin, showing PNI but no LVI. Three lymph nodes were identified, all negative for malignancy. However, extranodal deposits of ACC were observed, with the maximum tumor focus measuring 6 mm. The tumor was HER-2 negative and androgen receptor-positive (80%).

After a brief period, the patient presented with a left supraclavicular node (2 cm). Workup showed a pre-tracheal nodule of 5 mm. Bilateral central neck dissection was performed, and confirmed residual ACC with negative margins (the closest margin was 1 mm). No LVI or PNI was noted, and three reactive lymph nodes were identified, all negative for metastasis (0/3).

Follow-up PET scans two years after the end of radiotherapy showed a 5 mm nodule in front of the thyroid notch that was excised under local anesthesia, revealing recurrent ACC (8 mm in size) with free margins. Six months later (three years from diagnosis), a biopsy of a right pleural nodule confirmed metastatic ACC.

**Figure 2:**
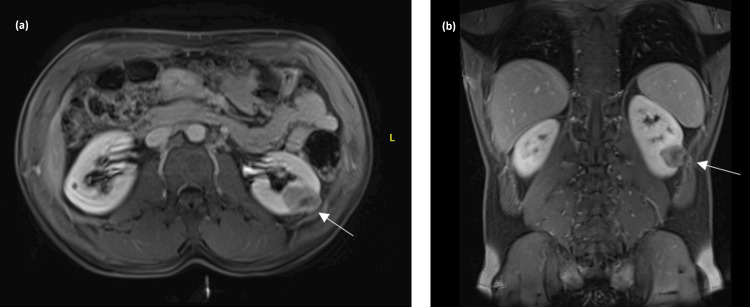
Abdominal MRI. (a) Axial T1 VIBE (volumetric interpolated breath-hold examination) and (b) coronal T1 VIBE showing a left renal exophytic lesion measuring 2.4 × 3.1 cm and demonstrating iso-/hyperintense T2 signal, isointense T1 signal, with diffusion restriction and faint enhancement, with no fat component or signal drop on in- and out-of-phase imaging.

Imaging showed a left renal lesion, described on abdominal MRI as an ill-defined, exophytic left kidney lesion measuring 2.4 × 3.1 cm, demonstrating iso-/hyperintense T2 signal, isointense T1 signal, with diffusion restriction and faint enhancement, with no fat component or signal drop on in- and out-of-phase imaging (Figure 2). There was no significant hydronephrosis or obstructive stone. The right kidney was normal without a suspicious lesion, apart from a 5 mm cyst in the right lower pole.

Since there was no other macroscopic site of disease, the patient underwent left partial nephrectomy, which was also confirmed to be metastatic ACC measuring 3.0 x 2.8 x 2.2 cm and invading the renal capsule into the perinephric fat, reaching Gerota's fascia surface and focally involving the parenchymal margin. Lymphovascular tumor emboli were also noted.

Disease progression thereafter was rapid, with the development of bilateral pleural, left perihilar, and bone metastases, in addition to local recurrence in the left renal operative bed.

Given the androgen receptor-positive nature of the tumor, the patient was started on bicalutamide. However, disease progression prompted a switch to abiraterone, prednisone, and goserelin.

The patient received palliative radiotherapy (20 Gy in five fractions) to the right chest wall, 4th right rib, and mediastinal lung metastasis.

Subsequently, the patient received three cycles of chemotherapy (cyclophosphamide, cisplatin, and doxorubicin), which resulted in a mixed response and slow progression of the left renal recurrence.

In total, he received nine cycles of chemotherapy, with overall disease progression. Thereafter, he received two lines of palliative chemotherapy: first with docetaxel, followed by Xeloda (capecitabine), during which continuous disease progression was observed along with the development of brain leptomeningeal metastasis.

Palliative whole-brain radiotherapy was offered; however, due to his poor general condition and the lack of additional suitable treatment options, his care was transitioned to the palliative care team. He passed away one month later.

## Discussion

ACC is an uncommon malignancy, representing approximately 1% of all head and neck cancers [1]. Although it most frequently originates in the salivary glands, ACC has been reported in a wide range of anatomical sites, including the nasal cavity, paranasal sinuses, palate, tongue, nasopharynx, external auditory canal, lacrimal glands, bronchus, lungs, breast, Cooper’s gland, skin, esophagus, vulva, cervix, and prostate [18,19].

ACC affects a wide age range, with the peak incidence typically occurring in the fifth to sixth decades of life. However, our patient was in his late 30s, which is younger than the typical presentation. The tumor exhibits a slight female predominance, with a female-to-male ratio of 3:2.

ACC is characterized by an indolent clinical course and has a tendency for delayed recurrence and metastasis after initial treatment. The most common metastatic sites are the lungs (80%), bones (14%), brain (3%), and skin (1%), with rarer involvement of organs such as the pancreas, thyroid, spleen, and breast [20,21]. Renal metastasis from ACC is extremely rare. The first case was described by Ladefoged et al. in 1984, involving a patient who developed kidney metastasis 23 years after undergoing pneumonectomy for ACC of the lung [6].

Due to the anatomical locations in which ACC arises, as well as postoperative fibrosis and structural distortion, clinical examinations and routine follow-up can be challenging. This makes early detection of recurrence or metastasis difficult. Furthermore, there are currently no standardized follow-up protocols for detecting metastases in the abdomen, including the kidneys, adding to the difficulty of managing these patients over the long term [22].

To the best of our knowledge, there have been only 13 reported cases of ACC metastasizing to the kidney (Table 1) [1,6-17]. Of the reported cases, 10 were females, and three were males, suggesting that renal metastasis from ACC may be more common in females. Nevertheless, this trend did not apply in our case, which involved a male patient.

**Table 1:** Clinicopathological features of 13 cases of metastases to the kidney from adenoid cystic carcinoma. M: male; F: female; ANED: alive with no evidence of disease; RAPN: robot-assisted partial nephrectomy.

Reference	Age (years)/sex	Clinical features	Primary tumor location/Size (cm)	Treatment of the primary tumor	Kidney metastasis time	Metastatic tumor location/Size (cm)	Treatment of metastatic tumor	Follow-up/outcome
Ladefoged et al. [6]	47/M	Hematuria	Between the middle and the lower right lung lobe/not reported	Right pneumonectomy	23 years	Left kidney/9.0 cm	Left radical nephrectomy	ANED after 12 months
Herzberg et al. [7]	57/F	Gross hematuria	Breast/1.5 cm	Modified radical mastectomy	12 years	Upper pole of the left kidney/6.5 cm	Left radical nephrectomy	ANED after 20 months
Blöchle et al. [8]	83/F	Incidental	Left lacrimal gland/not reported	Excision and radiotherapy	25 years	Left kidney/two small tumors	Left radical nephrectomy	Not mentioned
Manoharan et al. [9]	21/F	Incidental	Left parotid/not reported	Left parotidectomy and radiotherapy	7 years	Left kidney/9.0 cm	Left radical nephrectomy	ANED after 4 years
Vranic et al. [10]	76/F	Abdominal pain, hematuria, and urinary frequency	Breast/1.8 cm	Mastectomy	5 years	Upper part of the right kidney/9.0 cm	Right radical nephrectomy	Not mentioned
Santamaria et al. [11]	71/F	Not mentioned	Right palate/not reported	Right hemimaxillectomy	14 years	Right kidney/2.5 cm	Not reported	Not mentioned
Kala et al. [12]	35/F	Hematuria and right lumbar pain	Salivary gland/not reported	Surgical resection, chemotherapy, and radiotherapy	8 years	Upper pole of the right kidney/6.0 cm	Right radical nephrectomy	Not mentioned
Goyal et al. [13]	50/M	Flank pain	Lower lobe of the right lung/not reported	Right lower lobectomy, radiotherapy, and chemotherapy	7 years	Posterior of the right kidney/9.3 cm	Right radical nephrectomy	Not mentioned
Saha et al. [14]	28/F	Abdominal pain	Lower lobe of the right lung/8.1 cm	Chemotherapy (cisplatin and doxorubicin)	Meanwhile	Both kidneys/multiple lesions	Chemotherapy (cisplatin and doxorubicin)	3 months, died of disease
Qiu et al. [16]	26/M	Right waist pain	Left submandibular gland/not reported	Hemimaxillectomy and radiotherapy	3 years	Right kidney/6.0 cm	Right radical nephrectomy	Not mentioned
Bacalja et al. [15]	76/F	Hematuria and flank pain	Left lacrimal gland/not reported	Surgical resection	Lung metastases after 7 years, kidney metastases after 14 years	Lower pole of the right kidney/7.8 cm	Right radical nephrectomy	Not mentioned
Liu et al. [17]	65/F	Gross hematuria and left waist pain	Right parotid gland/2.5 cm	Surgical resection and radiotherapy	9 years	Lower pole of the left kidney/3.8 cm	Left radical nephrectomy and chemotherapy	ANED after 24 months
Piazza et al. [1]	58/F	Left flank pain	Right lung	Lobectomy and radiotherapy	14 years	Left kidney/7.5 cm Right kidney/1 cm	Bilateral RAPN with a two-stage approach	ANED after 12 months

The primary tumor sites among the reported cases were diverse. Four originated in the lung, two in the breast, two in the lacrimal gland, two in the parotid gland, one in the right palate, one in the left submandibular gland, and one in an unspecified salivary gland [1,6-17]. These findings highlight the wide anatomical distribution of ACC and its potential to metastasize to the kidney from multiple primary sites.

Among these cases, metastases to the kidney originated from various sites such as the breast, lung, auditory canal, and lacrimal gland. Moreover, kidney metastasis in these cases developed after varying periods, ranging from three to 14 years.

A single reported case described a primary pulmonary ACC in a 28-year-old female who presented with metastases to the liver and both kidneys simultaneously [1]. Although limited data suggest that renal metastasis may occur years after surgical resection of the primary ACC, our case involved metastasis occurring three years from the diagnosis and less than a year after completion of radical treatment of the recurrent disease at the primary site.

Surgery and radiotherapy are the main treatments for ACC, irrespective of the primary site. Among the reported cases, five patients underwent surgical excision of the primary tumor, five received both surgical resection and radiotherapy, two were treated with surgical resection, radiotherapy, and chemotherapy, and one received chemotherapy alone.

In contrast, the management of kidney metastases from ACC remains poorly defined. Up to now, surgery has been the main treatment method proposed for kidney metastases [16], which was the case in this report. Alternative treatment options, such as thermo-ablation or renal embolization, may be considered, particularly for painful lesion sites [23].

Systemic chemotherapy using agents like cyclophosphamide, cisplatin, 5-fluorouracil (5-FU), and doxorubicin may be effective against metastatic ACC and should be considered when locoregional treatments are not indicated.

In conclusion, we report a case of renal metastasis occurring three years after local treatment of the left submandibular gland ACC. Given the rarity of kidney metastasis from ACC, more cases should be documented to further understand its epidemiological, clinical, and imaging features, molecular genetic characteristics, and pathogenesis.

## Conclusions

This case highlights the extreme rarity of renal metastasis from ACC of the submandibular gland and reflects the unpredictable clinical course of this malignancy. Despite its typically indolent behavior, ACC can metastasize to unusual sites, even within a relatively short period following treatment. Clinicians should maintain a high index of suspicion for atypical metastatic presentations during follow-up, particularly when new clinical or radiological findings arise. The absence of standardized surveillance protocols, especially for abdominal involvement, remains a challenge and may contribute to delayed diagnosis. Given the limited evidence available, management of renal metastasis from ACC is not well established and should be individualized within a multidisciplinary setting. Reporting additional cases is essential to improve understanding of disease patterns and to help guide future diagnostic and therapeutic strategies.
